# Genome-wide DNA methylation analysis of pediatric medulloblastomas from a Brazilian cohort: an exploratory study

**DOI:** 10.1007/s12094-026-04304-w

**Published:** 2026-03-02

**Authors:** Hadassa G. Ortiz, Ciliana Rechenmacher, William B. Domingues, Antônio Duarte Pagano, Frederico Schmitt Kremer, Antônio Fernando da Purificação Júnior, Sidnei Epelman, Ricardo Fernández-Ramires, Sebastian Morales-Pison, Mariana B. Michalowski, Vinicius F. Campos

**Affiliations:** 1 Laboratório de Genômica Estrutural, Programa de Pós-Graduação em Biotecnologia, Centro de Desenvolvimento Tecnológico, Universidade Federal de Pelotas, Campus Universitário Capão do, Leão s/no - Prédio 20, Jardim América, Capão do Leão, Rio Grande do Sul 96010-900 Brazil; 2 Laboratório de Pediatria Translacional, Serviço de Pesquisa Experimental, Hospital de Clínicas de Porto Alegre, Porto Alegre, Brazil; 3 Laboratório de Bioinformática—Omixlab, Centro de Desenvolvimento Tecnológico, Universidade Federal de Pelotas, Capão do Leão, Rio Grande do Sul, Brazil; 4 Faculdade Santa Marcelina, Departamento de Oncologia Pediátrica, Casa de Saúde Santa Marcelina, São Paulo, São Paulo, Brazil; 5 Grupo Chileno de Cancer Herditario (GCCH), Santiago, Chile; 6 Facultad de Medicina y Ciências de La Salud, Universidad Mayor, Santiago, Chile

**Keywords:** Medulloblastoma, Molecular stratification, DNA methylation, Pediatric oncology, Epigenetics, Molecular profiling

## Abstract

**Background:**

DNA methylation profiling is a central tool for molecular classification of pediatric medulloblastoma. However, most reference datasets are derived from high-income countries, and the performance of established epigenetic markers in underrepresented populations remains insufficiently explored.

**Methods:**

We performed an exploratory genome-wide DNA methylation analysis of 15 pediatric medulloblastomas from a Brazilian cohort using the Illumina Infinium MethylationEPIC v2.0 array. Differentially methylated regions were identified using FDR-corrected statistical analyses and evaluated across molecular subgroups (SHH, Group 3, and Group 4). Functional annotation was conducted to assess biological themes associated with subgroup-enriched methylation patterns.

**Results:**

Methylation profiling revealed largely overlapping epigenetic landscapes among subgroups, alongside a limited number of subgroup-associated differentially methylated regions. These regions were mainly located in regulatory genomic elements and involved genes related to transcriptional regulation and developmental pathways. Several findings were consistent with previously reported subgroup-associated features, while additional loci not commonly included in diagnostic panels were observed.

**Conclusion:**

This exploratory study highlights the importance of validating DNA methylation–based biomarkers across diverse populations. The global methylation patterns observed in this Brazilian cohort were largely consistent with those reported in large international reference datasets, while also underscoring the value of population-aware analyses in expanding the diversity of epigenomic reference data.

**Supplementary Information:**

The online version contains supplementary material available at 10.1007/s12094-026-04304-w.

## Introduction

Medulloblastoma is the most common malignant brain tumor of childhood, accounting for approximately 20% of pediatric central nervous system neoplasms worldwide and representing a major cause of cancer-related morbidity and mortality in this age group [1]. Advances in molecular biology over the past decade have reshaped the understanding of medulloblastoma, leading to its classification into four major molecular subgroups—WNT, SHH, Group 3, and Group 4—each characterized by distinct genetic, epigenetic, and clinical features [2–4]. This molecular stratification has become central to risk assessment, prognostic evaluation, and therapeutic decision-making.

DNA methylation profiling has emerged as one of the most robust tools for medulloblastoma classification. Large-scale studies have demonstrated that methylation signatures provide highly reproducible subgroup discrimination and capture biological heterogeneity not fully explained by histopathology or transcriptomics alone [5–7]. DNA methylation profiling has become an established tool for the molecular classification of medulloblastoma and other central nervous system tumors, forming the basis of several widely adopted reference classifiers and stratification frameworks, and significantly improving diagnostic accuracy while being increasingly incorporateed into routine neuropathological practice [8]. In parallel, DNA methylation profiling has enabled the identification of subgroup-specific epigenetic programs, contributing to the understanding of tumor biology, clinical behavior, and therapeutic vulnerability.

Despite these advances, most reference datasets used to train and validate methylation classifiers originate from European and North American cohorts. Consequently, the extent to which these epigenetic signatures fully represent tumors arising in underrepresented populations remains poorly explored. Emerging evidence suggests that population-specific genetic backgrounds, environmental exposures, and healthcare contexts may influence epigenetic landscapes, potentially affecting biomarker performance and interpretation [9–11]. In Brazil, where pediatric medulloblastoma outcomes remain inferior to those reported in high-income countries [10], comprehensive molecular profiling studies remain scarce, particularly those addressing DNA methylation patterns.

DNA methylation is a dynamic and context-dependent epigenetic modification involved in transcriptional regulation, chromatin organization, and cellular differentiation. Aberrant methylation patterns—especially within differentially methylated regions (DMRs)—have been associated with tumor initiation, progression, and therapeutic response across multiple cancer types, including medulloblastoma [12–15]. However, the functional interpretation of methylation changes remains complex, as gene body and promoter methylation may exert distinct and sometimes opposing effects on gene expression, a phenomenon commonly referred to as the “DNA methylation paradox” [16]. Consequently, methylation-based findings must be interpreted cautiously, particularly in the absence of matched transcriptomic data.

Previous studies have identified subgroup-associated methylation patterns involving genes, such as CDKN2A, RASSF1, PTCH1, and OTX2, among others [13–15, 17, 18]. Nevertheless, most of these analyses have relied on large international cohorts, and limited data are available regarding how these epigenetic signatures behave in populations that are underrepresented in global datasets. Furthermore, while several diagnostic gene panels based on expression or methylation have been proposed [19], their performance and reproducibility across diverse populations remain insufficiently explored.

In this context, the present study aimed to perform an exploratory genome-wide DNA methylation analysis of pediatric medulloblastomas from a Brazilian cohort using the Illumina EPIC platform. Rather than proposing novel diagnostic markers or developing classification models, this work seeks to characterize subgroup-associated methylation patterns, evaluate the distribution of differentially methylated regions across genomic features, and assess how commonly used diagnostic genes behave within this population. By providing a population-aware epigenetic overview from a genetically diverse and historically underrepresented Brazilian cohort, this study aims to contribute to ongoing efforts to refine molecular classification frameworks and highlights the importance of inclusive reference datasets for precision oncology. From a clinical perspective, this population-aware epigenetic characterization is particularly relevant for contextual interpretation of molecular data in settings with limited access to comprehensive diagnostics, where DNA methylation profiling may complement existing approaches rather than serve as a standalone diagnostic tool.

## Materials and methods

### Study design and ethical approval

This study was designed as an exploratory, retrospective epigenomic analysis aimed at characterizing DNA methylation patterns in pediatric medulloblastomas from a Brazilian cohort. The primary objective was to evaluate subgroup-associated methylation profiles and assess the behavior of previously reported diagnostic loci in an underrepresented population.

All tumor samples were obtained from a reference pediatric oncology center in Brazil. The study was conducted in accordance with the Declaration of Helsinki and approved by the Institutional Research Ethics Committee (CAAE 29227020.6.1001.0066). Written informed consent was obtained from the legal guardians of all patients prior to sample inclusion.

### Sample selection and clinical characterization

Fifteen pediatric medulloblastoma cases were initially included based on the following criteria: histopathological confirmation of medulloblastoma; availability of suficient formalin-fixed paraffin-embedded (FFPE) tissue; adequate DNA quality for methylation analysis; availability of basic clinicopathological data. Tumors were previously classified according to histopathological and molecular criteria following WHO CNS5 recommendations. WNT medulloblastomas were represented by a single case in the cohort and, therefore, were not included in subgroup-specific methylation analyses due to the lack of biological replication. Clinical variables, including age at diagnosis, sex, tumor subgroup, and treatment modality, are summarized in Supplementary Table S1. Given the limited cohort size, no survival or outcome-based analyses were performed.

### DNA extraction and quality assessment

Genomic DNA was extracted from 10-µm FFPE tissue sections using the ReliaPrep™ FFPE gDNA Miniprep System (Promega, USA), following the manufacturer’s protocol. DNA concentration was quantified using a Qubit 4 Fluorometer (Thermo Fisher Scientific), and DNA integrity was assessed using the Illumina FFPE QC Kit. Samples presenting Δ*C*_q_ ≤ 5 were considered suitable for downstream processing, in accordance with Illumina quality guidelines. All samples included in the analysis met these criteria.

### Bisulfite conversion and array hybridization

A total of 250 ng of genomic DNA per sample was subjected to bisulfite conversion using the EZ DNA Methylation-Gold™ Kit (Zymo Research). Following conversion, DNA restoration was performed using the Infinium® FFPE DNA Restore Kit to ensure compatibility with array hybridization. Genome-wide DNA methylation profiling was conducted using the Illumina Infinium MethylationEPIC v2.0 BeadChip, which interrogates over 850000 CpG loci across regulatory and gene-associated regions of the human genome. Arrays were scanned using the Illumina iScan System according to the manufacturer’s protocols.

### Data preprocessing and quality control

Raw IDAT files were processed using the Minfi Bioconductor package (v1.14.0). Quality control procedures included evaluation of signal intensity distributions, detection p values, and inspection of control probes to ensure overall data integrity. Probes were excluded if they presented detection *p* values greater than 0.01 in more than 5% of samples, mapped to sex chromosomes, or contained known cross-reactive sequences or single-nucleotide polymorphisms that could interfere with probe hybridization. Data normalization was performed using the Subset-quantile Within Array Normalization (SWAN) method to minimize technical variability while preserving biological signal [20].

### Definition of differentially methylated regions (DMRs)

Differential methylation analysis was performed at the CpG level and summarized at the regional level. In this study, differentially methylated regions (DMRs) were defined exclusively based on subgroup-associated methylation differences identified through within-cohort comparisons among SHH, Group 3, and Group 4 tumors. Due to the absence of matched normal cerebellar tissue or paired non-tumor samples, DMRs were interpreted as relative differences in methylation patterns among molecular subgroups rather than as tumor-acquired epigenetic alterations. A DMR was defined as a genomic region containing at least two CpG probes exhibiting a consistent direction of methylation change, an absolute methylation difference (|Δ*β*|) ≥ 0.20, and a false discovery rate-adjusted *p* value < 0.05 using the Benjamini–Hochberg correction. DMRs were annotated according to genomic context, including promoter regions (TSS200 and TSS1500), gene body, 5′ untranslated region (5′UTR), 3′ untranslated region (3′UTR), and intergenic regions. Regions were classified as hypermethylated when Δ*β* > 0.20 and hypomethylated when Δ*β* <−0.20. Accordingly, the proportion of shared differentially methylated genes observed across subgroups reflects overlap in relative methylation differences identified through subgroup-to-subgroup comparisons, rather than absolute epigenetic similarity or clinical equivalence among tumors.

### Subgroup-based methylation analysis

Differential methylation analyses were performed independently for SHH, Group 3, and Group 4 tumors. Given the limited sample size, analyses were conducted in an exploratory manner and were not intended for classifier development or prognostic inference. To evaluate subgroup specificity, genes were categorized as shared among all subgroups, shared between two subgroups, or unique to a single subgroup. Overlap analyses were performed at the gene level to reduce probe-level noise and enhance biological interpretability.

### Analysis of clinically relevant diagnostic genes

Genes previously proposed for medulloblastoma molecular classification based on expression-based assays were evaluated for methylation patterns across genomic regions. This analysis aimed to assess whether methylation profiles at these loci were consistent across molecular subgroups or exhibited subgroup-specific variability. This analysis was not intended to validate or replace established diagnostic tools but rather to explore the epigenetic behavior of commonly used diagnostic markers within this cohort and to evaluate potential population-related variability.

### Functional enrichment analysis

Functional enrichment analyses of subgroup-specific genes were performed using Gene Ontology (GO) and the PANTHER classification system. Enrichment was assessed using Fisher’s exact test with false discovery rate correction. Given the exploratory nature of the study and the absence of transcriptomic data, enrichment results were interpreted descriptively and were not used to infer causal biological mechanisms.

### Statistical analysis

All statistical analyses were conducted using R software (version 4.3). False discovery rate correction was applied to all differential methylation analyses using the Benjamini–Hochberg method. No predictive modeling, survival analysis, or classifier training was performed due to the limited sample size. Results are therefore presented as hypothesis-generating and intended to support future validation studies.

### Data availability

Due to ethical restrictions related to patient confidentiality, raw methylation data are not publicly available. De-identified datasets may be made available upon reasonable request and subject to approval by the institutional ethics committee.

## Results

### Quality control and global methylation profile

All fifteen FFPE-derived medulloblastoma samples included in this study passed the quality control criteria required for DNA methylation analysis. DNA integrity and bisulfite conversion efficiency were confirmed using the Illumina FFPE QC workflow, with all samples presenting Δ*C*_q_ values ≤ 5. After preprocessing and normalization using the SWAN method, probe signal distributions and control metrics demonstrated consistent performance across samples, indicating adequate technical quality for downstream analyses.

Following probe filtering and normalization, high-quality methylation profiles were obtained for all samples, allowing reliable comparative analyses between molecular subgroups.

### Overview of differential methylation across medulloblastoma subgroups

Subgroup-specific DNA methylation analyses were performed for SHH and Group 3/4 tumors. A large number of differentially methylated regions (DMRs) were identified in each subgroup, reflecting the extensive epigenetic remodeling characteristic of medulloblastoma. As summarized in Table 1, most differentially methylated genes were shared among the three molecular subgroups. Approximately 92% of subgroup-associated differentially methylated genes identified through within-cohort subgroup comparisons were shared among SHH, Group 3, and Group 4 tumors, indicating a substantial overlap in relative methylation differences across non-WNT medulloblastomas.

**Table 1:** Summary of subgroup-associated differential methylation categories and corresponding gene counts across pediatric medulloblastoma subgroups

Subgroup	Differential methylation	*N* genes
G3	CDMR	118
	DMR	2887
	RDMR	182
G4	CDMR	118
	DMR	2889
	RDMR	179
SHH	CDMR	111
	DMR	2805
	RDMR	161

Genes are grouped according to the type of differentially methylated regions (DMRs) identified through within-cohort comparisons among SHH, Group 3, and Group 4 tumors. Values represent the number of genes associated with each methylation category per subgroup

**Fig. 1:**
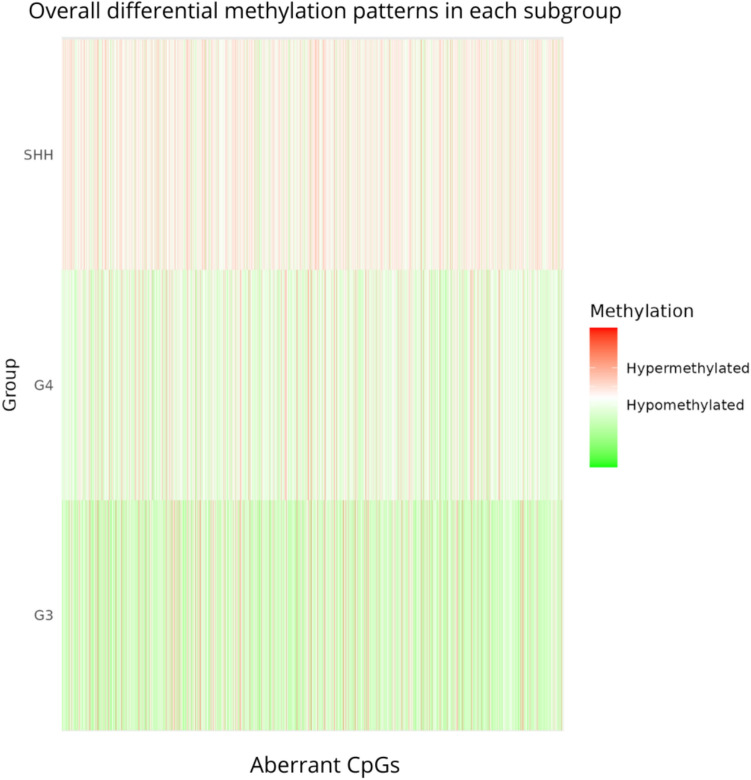
Heatmap showing subgroup-associated DNA methylation patterns in pediatric medulloblastomas. Columns represent CpG sites displaying statistically significant differential methylation identified through within-cohort comparisons among SHH, Group 3, and Group 4 tumors, and rows represent individual tumor samples. Color intensity reflects relative methylation differences, with green indicating hypomethylation and red indicating hypermethylation

The global methylation profile across samples is illustrated in Fig. 1, which shows the overall distribution of hypermethylated and hypomethylated CpG sites across all tumors. The heatmap highlights a heterogeneous methylation landscape, with both hypo- and hypermethylated regions present in all subgroups.

### Distribution of differentially methylated regions across genomic features

**Fig. 2:**
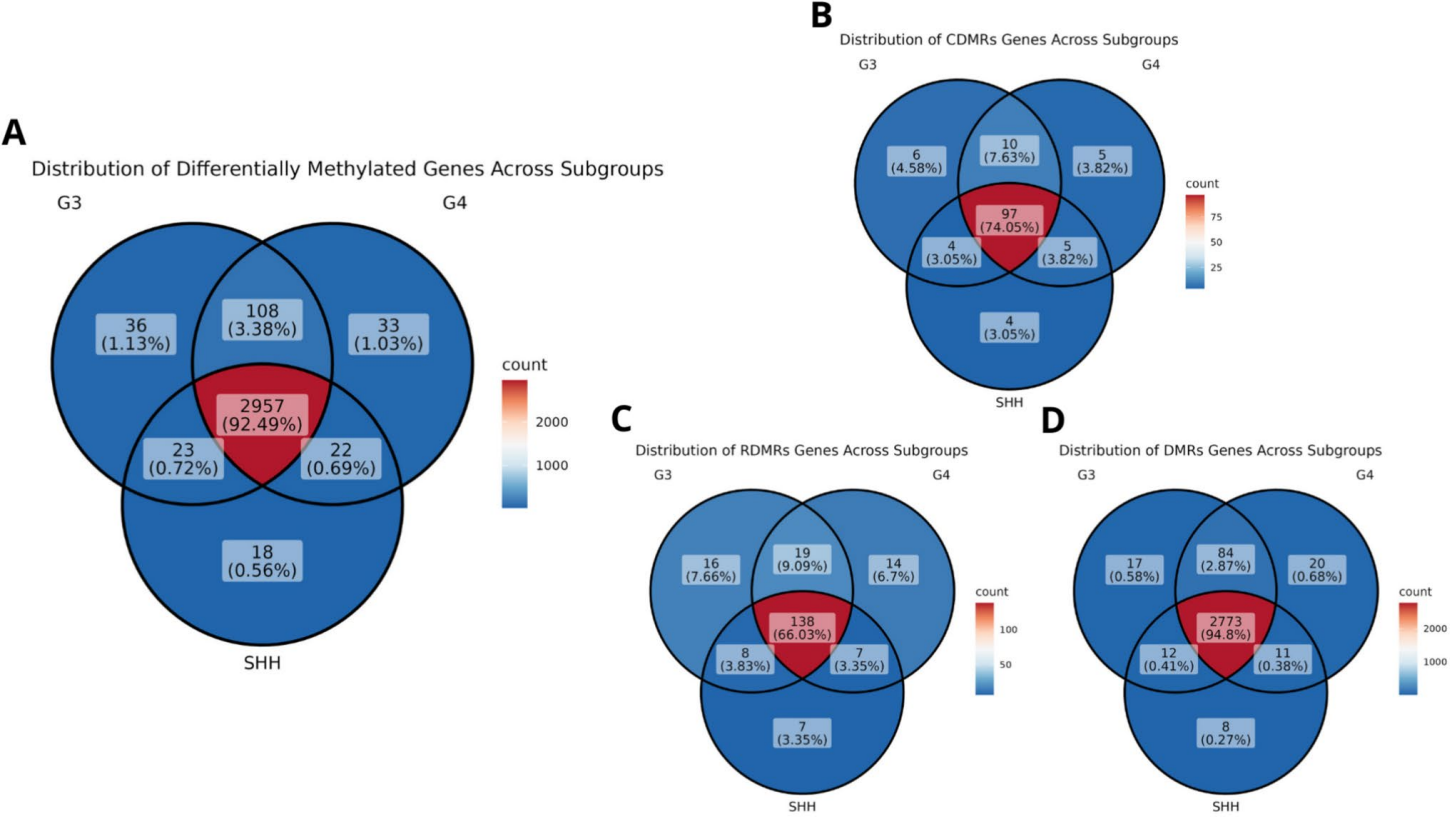
Venn diagrams illustrating the overlap and subgroup-specific distribution of differentially methylated genes among pediatric medulloblastoma subgroups. **A** Distribution of all subgroup-associated differentially methylated genes identified across SHH, Group 3, and Group 4 tumors. **B** Overlap and subgroup-specific distribution of genes associated with coding differentially methylated regions (CDMRs). **C** Distribution of genes associated with regulatory differentially methylated regions (RDMRs). **D** Distribution of genes associated with differentially methylated regions (DMRs) irrespective of genomic annotation. Percentages indicate the proportion of genes shared among subgroups or unique to a given subgroup based on within-cohort comparisons

Analysis of the genomic distribution of DMRs revealed that methylation changes occurred predominantly in regulatory regions rather than coding exons. Across all subgroups, the majority of DMRs were located in promoter-associated regions (TSS200 and TSS1500), followed by gene bodies and untranslated regions. This distribution pattern was consistent among SHH, Group 3, and Group 4 tumors and is depicted in Fig. 2, which illustrates the overlap of differentially methylated genes across subgroups and DMR categories. Although most genes were shared, each subgroup exhibited a limited number of unique differentially methylated genes, supporting the presence of subgroup-associated epigenetic variation.

### Subgroup-specific differentially methylated genes

To further characterize subgroup-associated methylation patterns, differentially methylated genes were stratified according to subgroup specificity. As shown in Fig. 2A–D, SHH, Group 3, and Group 4 tumors each displayed a small subset of uniquely methylated genes, while the majority of genes overlapped among groups.

In SHH tumors, differentially methylated genes were predominantly associated with regulatory regions and showed a balanced distribution between hypermethylation and hypomethylation. Group 3 tumors exhibited a higher density of DMRs in promoter and untranslated regions, suggesting a stronger involvement of regulatory elements. In contrast, Group 4 tumors displayed a more heterogeneous methylation pattern, with a greater proportion of hypomethylated regions distributed across multiple genomic contexts.

Despite these subgroup-specific trends, the relatively small number of exclusive genes compared to the large shared fraction indicates that epigenetic differences between subgroups are subtle and occur within a largely conserved methylation framework.

### Methylation profiles of clinically relevant diagnostic genes

The methylation status of genes commonly employed in molecular classification panels for medulloblastoma was specifically evaluated to assess how their epigenetic profiles behave in this cohort. These genes were selected based on previously published expression-based diagnostic assays, particularly the real-time PCR panel proposed by Kunder et al. [19], which relies on differential gene expression rather than DNA methylation status for subgroup assignment. The present analysis therefore aimed to investigate whether these clinically relevant genes also exhibit consistent methylation patterns that could support or complement expression-based classification approaches.

**Fig. 3:**
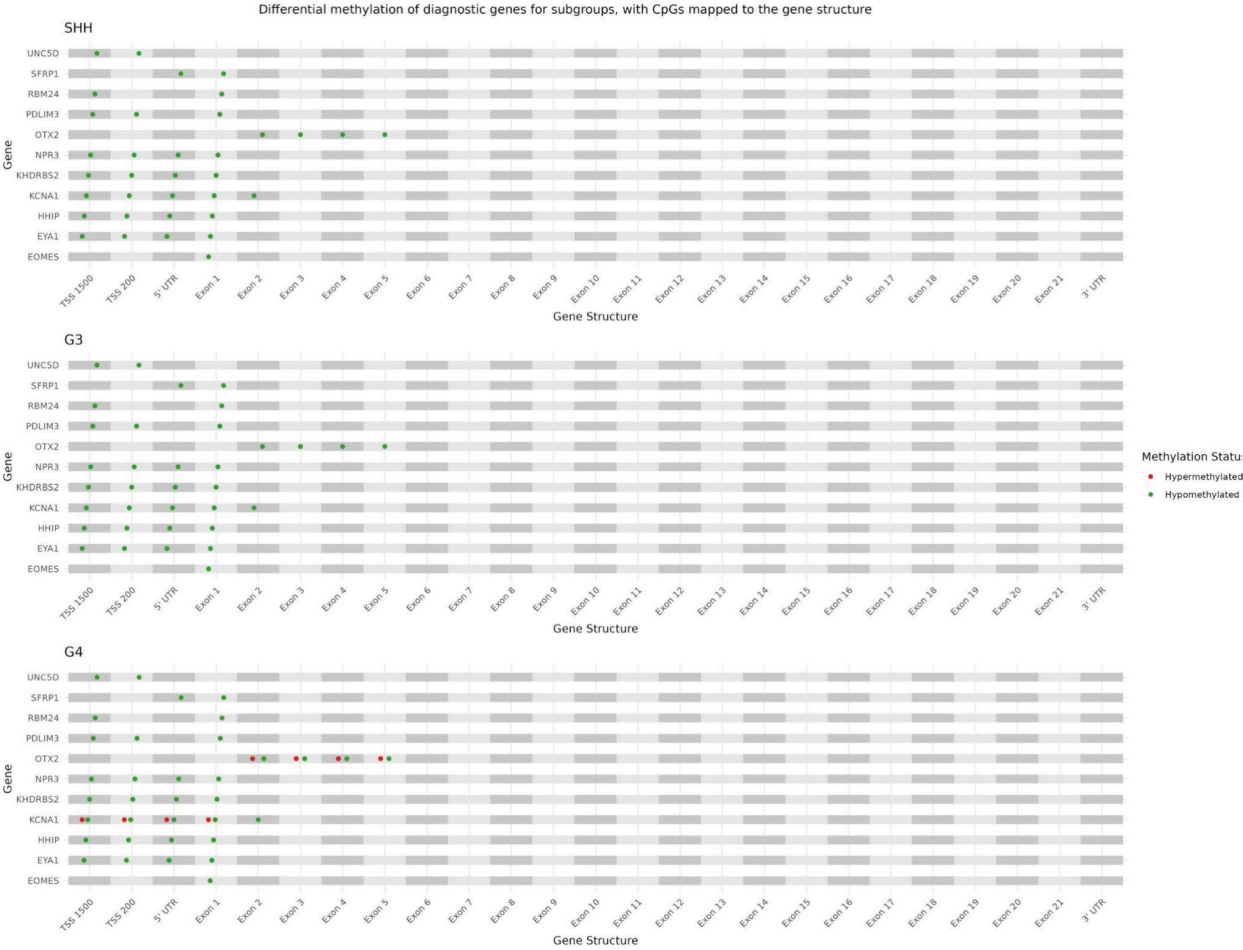
Subgroup-associated DNA methylation patterns of genes commonly used for molecular classification of medulloblastoma. CpG sites were mapped to distinct gene features across SHH, Group 3 (G3), and Group 4 (G4) tumors, including promoter regions, 5′ untranslated regions (5′UTRs), exons, and 3′ untranslated regions (3′UTRs). Each row corresponds to an individual gene, and each column represents a specific gene feature. Dots indicate CpG sites exhibiting statistically significant differential methylation identified through within-cohort subgroup comparisons, with red indicating hypermethylation and green indicating hypomethylation. This analysis is descriptive and does not imply direct correspondence between methylation status and gene expression

As shown in Fig. 3 and summarized in Table 2, the analyzed diagnostic genes displayed heterogeneous methylation profiles across subgroups and across different genomic regions. While some genes exhibited relatively consistent methylation patterns, no uniform methylation signature capable of clearly discriminating SHH, Group 3, and Group 4 tumors was observed. Importantly, most methylation changes were located within gene bodies or untranslated regions rather than promoter regions, which limits direct inference of transcriptional regulation based solely on methylation status.

**Table 2:** Genes commonly recommended by the world health organization (WHO) for molecular stratification of medulloblastoma subgroups and their expected subgroup association based on SYBR Green real-time quantitative PCR (RT-qPCR) assays, as previously described [18]

Subgroup	Expected genes and their regulation in the subgroup
SHH	↑ HHIP
	↑ MYCN
	↑ EYA1
	↑ PDLIM3
	↓ OTX2
	ATOH1
	SFRP1
G3	↑ OTX2
	↑ EOMES
	↑ NPR3
	↑ IMPG2
	↑ MYC
	↑ EGFL1L
	↓ GRM8
	↓ UNC5D
	GABRA5
	NRL
	MAB21L2
G4	↑ OTX2
	↑ EOMES
	↓ NPR3
	↓ IMPG2
	↓ MYC
	↑ MYCN
	↑ GRM8
	↑ UNC5D
	↑ KCNA1
	KHDRBS2
	RBM24
	OAS

Genes are listed according to their reported diagnostic use in expression-based stratification frameworks. This table summarizes reference information and does not represent diagnostic validation within the present cohort

This finding is particularly relevant in light of the fact that the diagnostic panel described by Kunder et al. [19] is based on gene expression measured by quantitative PCR, rather than epigenetic regulation. The lack of a direct correspondence between methylation status and subgroup classification observed in this study is therefore not unexpected and is consistent with the well-described context-dependent relationship between DNA methylation and transcriptional activity. In particular, gene body methylation has been shown to exhibit variable associations with gene expression depending on cellular context, tumor type, and chromatin architecture.

Taken together, these results indicate that although the genes included in expression-based diagnostic panels are robust markers at the transcriptomic level, their methylation profiles do not necessarily mirror subgroup identity in a consistent or predictive manner. This observation reinforces the notion that methylation and expression data represent complementary but non-equivalent layers of molecular regulation. Consequently, methylation profiling alone may not be sufficient to reproduce expression-based classification schemes, especially in small or population-specific cohorts.

These findings further emphasize the importance of interpreting methylation data within an integrated multi-omics framework and highlight the need for caution when extrapolating diagnostic or biological conclusions from methylation data in isolation.

### Subgroup-specific methylation patterns in unique genes

**Fig. 4:**
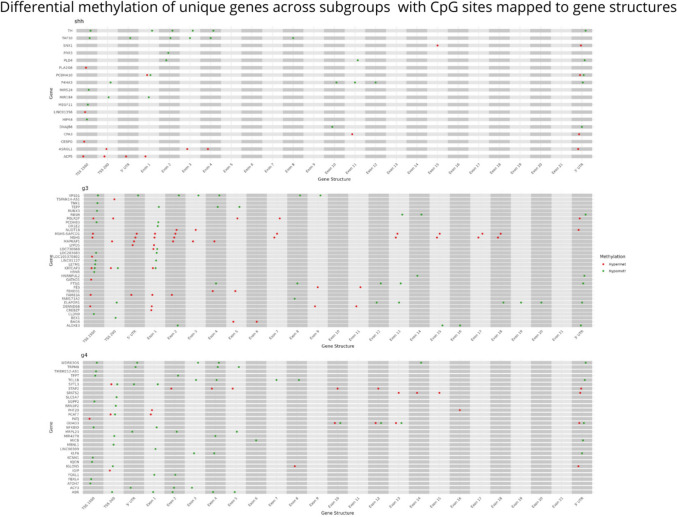
Subgroup-specific DNA methylation patterns of genes uniquely associated with medulloblastoma molecular subgroups. CpG sites mapped to distinct gene features are shown for genes identified as unique to SHH, Group 3 (G3), or Group 4 (G4) tumors based on within-cohort comparisons. Gene features include promoter regions, 5′ untranslated regions (5′UTRs), exons, and 3′ untranslated regions (3′UTRs). Each row corresponds to an individual gene, and each column represents a specific gene feature. Dots represent CpG sites exhibiting statistically significant differential methylation, with red indicating hypermethylation and green indicating hypomethylation. This representation is descriptive and does not imply direct functional or transcriptional consequences

The distribution of differentially methylated CpG sites within subgroup-specific genes is illustrated in Fig. 4. In SHH tumors, unique genes showed a predominance of regulatory-region methylation, with both hypermethylated and hypomethylated sites observed. Group 3 tumors exhibited a higher density of regulatory-region methylation events, particularly within promoter and 5′ untranslated regions. In Group 4 tumors, methylation changes were more frequently hypomethylated and distributed across diverse genomic regions.

These patterns indicate that although subgroup-specific methylation signatures exist, they are characterized by heterogeneous genomic localization and do not converge on a single regulatory mechanism.

### Functional enrichment analysis

Functional enrichment analysis of subgroup-specific differentially methylated genes is summarized in Table S1. Across all subgroups, enriched biological processes were primarily related to transcriptional regulation, signal transduction, cellular organization, and protein modification.

SHH-associated genes were enriched for pathways related to transcriptional control, extracellular matrix organization, and protein modification. Group 3 tumors showed enrichment for pathways associated with signaling, RNA processing, and immune-related functions. Group 4 tumors were enriched for genes involved in chromatin organization, ion transport, and cellular signaling.

Despite these subgroup-associated trends, substantial overlap in functional categories was observed, reinforcing the concept that methylation differences reflect modulation of shared biological processes rather than entirely distinct molecular programs.

### Summary of key findings

Collectively, the results demonstrate that pediatric medulloblastomas from this Brazilian cohort exhibit extensive DNA methylation alterations characterized by a large shared epigenetic core and a smaller subset of subgroup-associated differences. The predominance of regulatory-region methylation and the variability observed in clinically relevant genes highlight the complexity of interpreting methylation data in isolation.

These findings underscore the importance of population-aware analyses and support the need for integrative studies combining methylation, transcriptomic, and clinical data to refine molecular classification strategies in medulloblastoma.

## Discussion

DNA methylation has been widely recognized as an important epigenetic mechanism involved in tumor classification, prognosis, and therapeutic response in cancer, including pediatric brain tumors [21]. In the present study, we investigated genome-wide methylation profiles in pediatric medulloblastomas and observed that, although global methylation patterns differed among molecular subgroups, a substantial proportion of aberrantly methylated genes was shared across all groups. Specifically, 2,957 genes exhibited differential methylation in common among SHH, Group 3, and Group 4 tumors, while a smaller subset of genes showed subgroup-specific methylation patterns, including 18 genes in SHH, 36 in Group 3, and 33 in Group 4. The high proportion of subgroup-associated differentially methylated genes shared among SHH, Group 3, and Group 4 tumors should be interpreted considering the comparative design of this study. As differential methylation was defined based on within-cohort subgroup comparisons rather than against normal cerebellar tissue, this overlap reflects shared epigenetic features among non-WNT medulloblastomas rather than absolute epigenetic similarity or diagnostic equivalence. In this context, subgroup distinction arises primarily from the smaller subset of genes exhibiting subgroup-specific methylation patterns, which represent the principal source of biological divergence captured in this exploratory analysis. These findings highlight the limitations of global methylation overlap for stratification purposes while underscoring the value of subgroup-specific epigenetic features for hypothesis generation.

When evaluating genes currently used for molecular stratification by real-time qPCR, we observed largely similar methylation patterns across most gene regions in all subgroups, except for Group 4, which also exhibited hypermethylation in addition to hypomethylation. Clinically, this finding is relevant, because it reinforces that methylation-based data should not be interpreted as a surrogate for expression-based diagnostic assays, particularly in routine settings where therapeutic decisions rely on established subgroup classification frameworks. This finding is consistent with the well-established complexity of the relationship between DNA methylation and gene expression, often referred to as the “DNA methylation paradox,” in which methylation status does not necessarily correlate linearly with transcriptional activity [16]. Therefore, the relationship between DNA methylation and gene expression is context-dependent and should be interpreted cautiously, particularly in the absence of matched transcriptomic data. Given the limited cohort size, all findings should be interpreted as hypothesis-generating.

To better interpret subgroup-specific features, our discussion focused on genes displaying consistent patterns of exclusive hypermethylation or hypomethylation. Several genes previously described as epigenetically relevant in medulloblastoma, including *KLF4*, *PTCH1*, and *ZIC2*, were not identified as uniquely methylated in our cohort, highlighting the heterogeneity of epigenetic alterations across studies and populations [22, 23]. In contrast, hypermethylation of genes associated with the SHH subgroup, such as *SPINT2* and *RASSF1A*, was observed, in agreement with prior reports linking epigenetic regulation of these loci to tumor progression and metastatic behavior in medulloblastoma and other pediatric CNS tumors [2, 17]. Likewise, the diagnostic gene *OTX2*, commonly used in medulloblastoma classification, exhibited methylation patterns consistent with published data, including differential methylation in non-SHH tumors, although in our cohort, the aberrant CpG sites were not restricted to promoter regions, underscoring the complexity of epigenetic regulation at this locus [18].

Interpretation of subgroup-specific methylation patterns in this study is informed primarily by literature on medulloblastoma biology, neural development, and transcriptional regulation in central nervous system tumors. For several loci, direct functional evidence linking DNA methylation to transcriptional output in medulloblastoma remains limited. Therefore, the observed subgroup-associated methylation patterns are interpreted as hypothesis-generating and are considered within the context of CNS-specific regulatory networks rather than as direct mechanistic drivers. Recent integrative studies have demonstrated that epigenetic variation in medulloblastoma frequently converges on transcriptional regulators and developmental programs that define subgroup identity, supporting the biological plausibility of the patterns identified here [22, 24].

In SHH tumors, subgroup-specific methylation changes were observed in genes implicated in transcriptional regulation and neural developmental processes relevant to SHH-driven tumorigenesis. SHH medulloblastomas are known to arise from cerebellar granule neuron precursors and are characterized by distinct developmental trajectories and regulatory programs [23, 25]. Epigenetic modulation of genes involved in these pathways may reflect alterations in lineage commitment, proliferation, and differentiation rather than isolated gene-level effects. Consistent with this view, recent analyses of regulatory networks in SHH medulloblastoma have highlighted the central role of coordinated transcriptional and epigenetic control in shaping tumor behavior [24].

In Group 3 tumors, subgroup-specific methylation patterns affected genes associated with transcriptional regulation, cell cycle control, and progenitor-like cellular states. Group 3 medulloblastomas are characterized by aggressive clinical behavior and enrichment for MYC-driven transcriptional programs [2, 23]. Epigenetic alterations observed in this subgroup may therefore reflect broader dysregulation of regulatory networks that sustain proliferative capacity and impair differentiation, rather than direct effects on individual genes. These findings are consistent with previous molecular studies emphasizing the importance of epigenetic remodeling and transcriptional control in defining Group 3 tumor biology [24].

In Group 4 tumors, several differentially methylated genes identified in this study have been implicated in neuronal differentiation and transcriptional regulation within central nervous system contexts. Group 4 medulloblastomas are thought to originate from distinct cerebellar progenitor populations and to follow unique developmental trajectories compared with other subgroups [3]. Accordingly, subgroup-specific methylation patterns observed here may reflect epigenetic modulation of neural lineage-associated programs. Although functional validation is required, these patterns are compatible with current models of Group 4 tumor biology that emphasize developmental origin and regulatory network architecture [24].

The limited representation of WNT medulloblastomas represents an important limitation of this study. As WNT tumors constitute biologically and epigenetically distinct entities with markedly favorable clinical outcomes, the high degree of overlap observed in DNA methylation profiles should be interpreted as reflecting shared epigenetic features among non-WNT medulloblastomas (SHH and Group 3/4), rather than across all medulloblastoma subgroups. It is therefore possible that inclusion of WNT tumors would reduce the observed overlap and further emphasize subgroup-specific divergence.

Other important limitation of this study is the absence of matched normal cerebellar tissue or paired non-tumor samples, which precludes discrimination between tumor-acquired epigenetic alterations and tissue-specific methylation patterns intrinsic to the cerebellum. Consequently, the identified DMRs should be interpreted as reflecting relative methylation differences among medulloblastoma molecular subgroups rather than absolute deviations from normal cerebellar methylation. This limitation underscores the need for cautious biological interpretation and highlights the value of future studies integrating tumor–normal comparisons and multi-omic validation.

From a population perspective, this study contributes DNA methylation data from a Brazilian pediatric medulloblastoma cohort, a population that remains underrepresented in large international reference datasets. Although the global methylation patterns observed here were largely consistent with those reported in European and North American cohorts, subtle variations in subgroup-associated methylation profiles and locus-specific patterns were observed. These findings do not challenge the robustness of current methylation-based classification frameworks but rather underscore the value of expanding epigenomic reference datasets to include genetically diverse and admixed populations. Such population-aware analyses may be particularly relevant for refining the interpretation of subgroup-associated features and for contextualizing biomarker behavior across different genetic and environmental backgrounds.

Overall, these findings reinforce the concept that DNA methylation plays a multifaceted role in medulloblastoma biology, influencing tumor behavior, prognosis, and potentially therapeutic response. While genes commonly used for diagnostic stratification did not display uniform methylation signatures, subgroup-specific epigenetic alterations were identified and were consistent with previously reported cancer-related pathways. Importantly, our data support the notion that methylation-based analyses should be interpreted within a broader biological and clinical framework and not as standalone diagnostic tools.

In summary, this study demonstrates that pediatric medulloblastomas exhibit extensive and complex DNA methylation alterations, characterized by a large shared epigenetic background and a smaller set of subgroup-specific changes. The integration of these findings with the existing literature supports the relevance of methylation profiling as a complementary approach to molecular classiflication and highlights the importance of population-specific studies to refine precision oncology strategies in medulloblastoma.

## Conclusion

This study provides an exploratory and integrative view of DNA methylation patterns in a Brazilian cohort of pediatric medulloblastomas, highlighting both the shared epigenetic landscape across molecular subgroups and a limited set of subgroup-associated methylation differences. The findings of this study provide a comparative epigenetic overview across medulloblastoma subgroups rather than a tumor-versus-normal methylation framework. The predominance of common differentially methylated genes among SHH, Group 3, and Group 4 tumors reinforces the concept that medulloblastoma subtypes share a conserved epigenetic backbone related to neurodevelopmental and oncogenic processes. Accordingly, the conclusions of this study primarily apply to SHH and Group 3/4 medulloblastomas and should not be extrapolated to the WNT subgroup.

At the same time, the identification of subgroup-specific methylation patterns—particularly within regulatory regions—supports the existence of epigenetic modulation associated with tumor biology. However, the heterogeneous distribution of these alterations and their frequent localization outside promoter regions underscore the complexity of interpreting methylation data in isolation. The absence of a consistent correspondence between methylation status and expression-based diagnostic markers further emphasizes that DNA methylation and transcription represent complementary, but not interchangeable, regulatory layers.

Importantly, the subgroup-level methylation patterns observed in this Brazilian cohort are broadly consistent with those reported in large international series; thus, at a global level, we did not observe evidence suggesting major population-specific deviations in methylation subgroup signatures, although larger multi-population datasets will be required to test this formally [2, 11, 23]. Rather, subtle variations likely reflect biological heterogeneity, sample composition, and contextual factors inherent to epigenomic regulation. These findings reinforce the need for caution when extrapolating diagnostic or prognostic conclusions from methylation data alone, particularly in small or underrepresented cohorts.

Taken together, our results support the role of DNA methylation profiling as a valuable component of molecular characterization in medulloblastoma, while also highlighting its limitations when used in isolation. The integration of methylation data with transcriptomic, genomic, and clinical information in larger, multi-center cohorts will be essential to refine molecular classification frameworks and to improve the robustness and equity of precision oncology strategies in pediatric brain tumors.

## Supplementary Information

Below is the link to the electronic supplementary material.

Supplementary file1 (DOCX 20 kb)

## Data Availability

The datasets generated and analyzed during the current study are not publicly available due to patient confidentiality but are available from the corresponding author upon reasonable request and in compliance with ethical regulations.
