# Supplementary material for: Genome-wide DNA methylation analysis of pediatric medulloblastomas from a Brazilian cohort: an exploratory study
Source: Clin Transl Oncol. 2026 Mar 2;28(8):3461–72. doi: 10.1007/s12094-026-04304-w (PMC13401557; doi:10.1007/s12094-026-04304-w)
Supplement: Supplementary file 1 — Supplementary file1 (DOCX 20 kb) [file 12094_2026_4304_MOESM1_ESM.docx]

**Table S1** Stratification and summarization of differential methylation types with GO annotation for the unique subgroup genes

| Subgroup | Differential methylation type | Gene | Function family |
| --- | --- | --- | --- |
| SHH | CDMR | HIPK4; P4HA3 | Protein Modifying Enzymes |
|  | CDMR | TAF10 | General Transcription Factors |
|  | CDMR | TH | Oxidoreductases and Enzymes Related to Metabolism |
|  | DMR | PLA2G6; PLD4; ACP5 |  |
|  | DMR | MEGF11 | Extracellular Matrix Proteins   \|  \| \| --- \| |
|  | RDMR | CEBPG | Transcription and Regulatory Factors |
|  |  | DNAJB6 | Chaperones and Cytoskeletal Proteins |
|  |  | PCDHA10; PRR3; SNX1 | Cell Adhesion and Scaffold/Adaptor Proteins |
|  | RDMR | ASRGL1; CPA3 | Proteases and Metalloproteases |
|  | DMR | LINC01356;  MIR184 | Long Non-Coding RNA (lncRNA) and miRNA |
|  | RDMR | MIR524 |  |
| G3 | CDMR | FES | Protein Kinases & Signal Transduction Pathways |
|  | DMR | FAM83A |  |
|  | RDMR | TNK1; MAPKAP1 |  |
|  | CDMR | ALOXE3 | Oxygenases and Oxidative Stress Response |
|  | RDMR | NUDT18 |  |
|  | DMR | CREBZF; RUNX3; BEX1 | RNA and DNA Regulation (Transcription, Methylation, and RNA-related processes) |
|  | RDMR | POLR2F; FTSJ; GATAD1 |  |
|  | DMR | CLDN9; LETM1; OR1E2; LYPD5 | Transporters, Receptors, and Junction Proteins |
|  | RDMR | BAG6; HRNR | Chaperones and Cytoskeletal Proteins |
|  | RDMR | PCDHB3; FBXO31 | Cell Adhesion and Ubiquitination Pathways |
|  | DMR | LOC105370802 (lncRNA) | Apoptosis, Immune Function, and Cancer-Related Genes |
|  | RDMR | ELAPOR1; MSH5; MSH5-SAPCD1 (lncRNA); TSPAN14-AS1 (lncRNA) |  |
|  | DMR | LINC01127 (lncRNA) | Overexpressed in brain regions |
|  |  | LOC730668 (lncRNA) | Age-related |
|  |  | LOC283683 (lncRNA) | Tumorigenicity assay in ovarian cancer |
|  |  | VPS51; CCDC40; DENND6B | Golgi vesicle transport  Golgi organization  endocytic recycling  lysosomal transport  retrograde transport; endosome to Golgi; regulation of catalytic activity in the cytosol |
|  |  | FAM171A2;  KRTCAP3 | Predicted to be integral component of membrane |
|  | RDMR | RBSN | Endocytic membrane fusion and membrane trafficking of recycling endosomes |
| G4 | CDMR | ATOH7 | Basic helix-loop-helix transcription factor |
|  | CDMR | KLF6 | C2H2 zinc finger transcription factor |
|  | RDMR/DMR | IGLON5 | Immunoglobulin |
|  | RDMR | IGIP |  |
|  | DMR | SLC5A7 | Ion channel |
|  | RDMR | MICB | Major histocompatibility complex protein |
|  | DMR | SYTL3 | Membrane trafficking regulatory protein |
|  | RDMR | MBNL1 | RNA splicing factor |
|  | DMR | PATJ | Scaffold/adaptor protein |
|  | RDMR | STAP2 |  |
|  | RDMR | KCNN1 | Voltage-gated ion channel |
|  | DMR | FOXL1 | Winged helix/forkhead transcription factor |
|  | CDMR | TMEM212-AS1 | Long non-coding RNA |
|  | RDMR | PCAT7 |  |
|  | DMR | LINC00391; LINC00309 |  |
|  | CDMR | TCL1B | None specified |
|  | RDMR | WDR83OS | Enables protein folding chaperone. Involved in protein insertion into ER membrane. |
|  |  | FBXL4 | Class I MHC mediated antigen processing and presentation and Metabolism of proteins |
|  |  | PHF20 | H4K5 acetyltransferase activity and histone H4K16 acetyltransferase activity |
|  |  | SPATS2 | RNA binding |
|  |  | IQCN | Essential for spermiogenesis and fertilization |
|  |  |  |  |
|  | DMR | ACY3 | Identical protein binding and hydrolase activity, acting on carbon-nitrogen |
|  |  | TRPM8 | protein  Homodimerization activity and calcium channel activity. |
|  | DMR | ODAD3 | enables protein  binding |
|  | DMR | TFPT | protein  heterodimerization activity and protein kinase binding |
|  | DMR | C4orf50 | Chromosome 4 Open Reading Frame 50 |
